# Tumor cell response to bevacizumab single agent therapy *in vitro*

**DOI:** 10.1186/1475-2867-13-94

**Published:** 2013-09-23

**Authors:** Melanie Hein, Shannon Graver

**Affiliations:** 1 Developmental Biochemistry, Biocenter, University of Wuerzburg, Wuerzburg, Germany; 2 Physiological Chemistry, Biocenter, University of Wuerzburg, Wuerzburg, Germany

**Keywords:** Bevacizumab, NCI-60, Tumor angiogenesis, VEGFA, Hypoxia, *In vitro*

## Abstract

**Background:**

Angiogenesis represents a highly multi-factorial and multi-cellular complex (patho-) physiologic event involving endothelial cells, tumor cells in malignant conditions, as well as bone marrow derived cells and stromal cells. One main driver is vascular endothelial growth factor (VEGFA), which is known to interact with endothelial cells as a survival and mitogenic signal. The role of VEGFA on tumor cells and /or tumor stromal cell interaction is less clear. Condition specific (e.g. hypoxia) or tumor specific expression of VEGFA, VEGF receptors and co-receptors on tumor cells has been reported, in addition to the expression on the endothelium. This suggests a potential paracrine/autocrine loop that could affect changes specific to tumor cells.

**Methods:**

We used the monoclonal antibody against VEGFA, bevacizumab, in various *in vitro* experiments using cell lines derived from different tumor entities (non small cell lung cancer (NSCLC), colorectal cancer (CRC), breast cancer (BC) and renal cell carcinoma (RCC)) in order to determine if potential VEGFA signaling could be blocked in tumor cells. The experiments were done under hypoxia, a major inducer of VEGFA and angiogenesis, in an attempt to mimic the physiological tumor condition. Known VEGFA induced endothelial biological responses such as proliferation, migration, survival and gene expression changes were evaluated.

**Results:**

Our study was able to demonstrate expression of VEGF receptors on tumor cells as well as hypoxia regulated angiogenic gene expression. In addition, there was a cell line specific effect in tumor cells by VEGFA blockade with bevacizumab in terms of proliferation; however overall, there was a limited measurable consequence of bevacizumab therapy detected by migration and survival.

**Conclusion:**

The present study showed in a variety of *in vitro* experiments with several tumor cell lines from different tumor origins, that by blocking VEGFA with bevacizumab, there was a limited autocrine or cell-autonomous function of VEGFA signaling in tumor cells, when evaluating VEGFA induced downstream outputs known in endothelial cells.

## Introduction

Tumor cells are dependent on consistent oxygen and nutrient supply to promote tumor progression. Tumor cells co-opt new vessels from the existing host vascular network, driving tumor growth and the opportunity for metastatic spread [1].

Most solid tumors develop regions of low oxygen tension because of a tissue imbalance between oxygen supply and consumption [2]. Hypoxia inducible factor 1 (HIF-1) is one of the most important transcription factors of the hypoxic response in mammalian cells, regulating a multitude of biological processes including cell proliferation, cell migration, metabolism, apoptosis and angiogenesis. It thus acts on both the adaptation of affected cells and the improvement of their vascular supply. A well-studied hypoxia response in tumor cells is the production of growth factors that induce angiogenesis [3]. HIF-1 activates transcription of vascular endothelial growth factor (VEGFA), a major inducer of tumor angiogenesis. Signaling through its receptors VEGFR1 (FLT-1), VEGFR2 (FLK-1) and co-receptor Neuropilin1 (NRP1) on endothelia represents the best characterized pathway in angiogenesis [4].

In the 40 years since Judah Folkman first proposed the theory of targeting angiogenesis as a novel cancer therapy [5], anti-angiogenic treatment has found its way into clinical practice. The first approved therapeutic agent to specifically target the tumor associated vessels of solid tumors was bevacizumab, a monoclonal antibody against all isoforms of VEGFA [6].

Bevacizumab has proven efficacy combined with chemotherapy (or interferon in RCC) in clinical trials for metastatic colorectal cancer (CRC), non-small cell lung cancer (NSCLC), renal cell carcinoma (RCC) and metastatic breast cancer (BC) (reviewed in [7]) and received subsequent regulatory approval. The findings of many clinical trials and case studies detect an increase in response rates with the use of bevacizumab and/or a prolonged time until disease progression. However the impact on overall survival is more sporadic and not well defined.

Factors influencing response to bevacizumab treatment have been sought by the investigation of biomarkers to improve patient stratification (reviewed in [8]). One of the main pathways under investigation has been the VEGFA pathway itself. VEGFA acts on endothelial cells through its main receptor, VEGFR2, and is expressed at high levels at sites of neoangiogenesis in solid tumors [9].

There has been no consensus in literature on the expression of VEGF receptors in tumor tissue, especially whether they are found exclusively on endothelial cells or if tumor cells also benefit from VEGFA signaling via paracrine and/or autocrine signaling loops. While there is ample evidence for VEGF receptor expression on tumor vasculature [9,10], there are also several studies that demonstrate receptor expression on tumor cells themselves (NSCLC, CRC and BC) [11-21]. Inconsistencies seen with the use of anti-angiogenic therapy, led to the hypothesis that tumor cells may do more than just secrete a chemotactic agent for endothelial cells and may also contribute to response indicators seen clinically.

To investigate the potential effects of the VEGFA pathway in tumor cells, we employed a series of cell lines from the well-established NCI-60 panel to study angiogenic gene and protein expression. In addition, cellular responses were analyzed under both normoxia and hypoxia with reduced serum concentration, either with or without VEGFA blockade through bevacizumab. We showed that VEGF receptors are expressed by tumor cells and not only by endothelial cells, which highlights the prospect of complex angiogenic pathway/signaling cross talk between various cell types. By blocking a key regulator of the angiogenic pathway, VEGFA, our results did not show any adverse effects in tumor cells nor did bevacizumab alter the angiogenic potential of the VEGFA pathway in tumor cells. A functional consequence could be detected by a change in proliferation for one cell line in addition to the down regulation of *Neuropilin 1* in other cell lines. However, neither altered migration nor VEGF receptor 1 or 2 and ligand regulation was seen as a result of bevacizumab treatment.

## Material and methods

### Cell culture

Thirty human tumor cell lines selected from the NCI-60 panel (*NSCLC*: A549, EKVX, H226, H23, H460, H522, HOP62, HOP92, LXFL529; *CRC*: COLO205, HCT-116, HCT-15, HT-29, KM12, SW620; *RCC*: 786–0, A498, ACHN, CAKI-1, RXF-393, RXF-631, SN12C, SN12K1, TK-10, UO-31; *BC*: BT-549, HS-578 T, MDA-MB-231, MDA-MB-468, T47D) were obtained from American Type Culture Collection and cultured in RPMI-1640 supplemented with 10% FBS (PAN Biotech, Germany), 1% Glutamax (Gibco, Germany), 1% penicillin and 1% streptomycin (PAA, Austria) as recommended by the NCI-Frederick Cancer DCTD Tumor Cell Line Repository. Cells were passaged at ≈ 80% confluency.

HUVECs (human umbilical vein endothelial cells, a gift from A. Fischer, Mannheim as previously described [22] or purchased from PromoCell, Germany) were cultured in M199 medium (Sigma-Aldrich, Germany) with 10% FBS, 25 μg/ml heparin (Sigma-Aldrich, Germany), 50 μg/ml ECGS (Sigma-Aldrich, Germany) and 1% Glutamax on plates pre-coated with 0.2% gelatin. Reduced culture medium did not contain ECGS and serum concentration was reduced to 1% FBS.

Hypoxia experiments were performed at 1% O_2_ (Pro-Ox controller, Biospherix, USA) under serum-reduced conditions (NSCLC, CRC and RCC: 1% FBS; BC: 5% FBS). Where indicated, 50 ng/ml recombinant human VEGFA (Miltenyi Biotec, Germany) and 250 μg/ml bevacizumab (Avastin, was provided by Roche, Switzerland), was added.

### Cell proliferation assay

Cell proliferation was assessed for up to 96 hours using MTT [3-(4,5-dimethylthiazol-2-yl)-2,5-diphenyltetrazolium bromide] staining (Sigma-Aldrich, Germany) as previously described [23].

Briefly, between 2 × 10^3^ and 5 × 10^3^ cells/well (cell line/doubling time dependent) were seeded into 96 well plates and incubated overnight to adhere. Medium was then replaced by RPMI-1640 with reduced FBS and bevacizumab or VEGFA at the concentrations indicated (time point zero). After 24, 48, 72 or 96 hours in hypoxia, MTT (5 mg/ml in PBS) was added and incubated for 2 hours at 37°C. The supernatant was removed and reaction products were solubilized for 1 h in 10% HCl, 0.1% NP-40 in isopropanol. Absorbance was measured at 570 nm with a reference wavelength of 650 nm using an ELISA reader (Sunrise Absorbance Reader Tecan, Austria). Each experimental condition was analyzed in triplicate and results are an average of a minimum of three biological repetitions.

### Cell migration assay

Cell migration was measured using the *in vitro* scratch assay as described previously [24]. Briefly, cells were grown in 6 well plates to a confluent monolayer, then scraped in a straight line using a sterile P200 pipet tip in triplicate. To remove debris, cells were washed once with PBS. Medium was changed to serum reduced +/− bevacizumab and cells were incubated for up to 24 hours under hypoxia at 37°C. Images of the scratch width were measured using ImageJ software [25] at the same location after 6 and 24 hours of incubation.

### Cell lysis and immunoblot analysis

Cell pellets were lysed in lysis buffer (20 mM HEPES (pH 7.8), 500 mM NaCl, 5 mM MgCl_2_, 5 mM KCl, 0.1% sodium deoxycholate, 0.5% Nonidet-P40, 10 μg/ml Leupeptin, 10 μg/ml Aprotinin, 1 mM PMSF (phenylmethanesulphonylfluoride), 200 μM Na_3_VO_4_, 0.1 M NaF) for up to 4 hours on ice. Protein was resolved by SDS-polyacrylamide gel electrophoresis and analyzed by immunoblotting. The following antibodies were purchased from Santa Cruz Biotechnology (Heidelberg, Germany): anti-VEGFR1 (Flt1) (C17) rabbit, 1:200; anti-Neuropilin1 (H-286) 1:200. VEGFR2 1:200 and beta-Actin 1:10000 were purchased from Cell Signaling (MA, USA) Cleaved PARP 1:2000 was purchased from BD Bioscience (USA). Vinculin 1:10,000 was purchased from Sigma-Aldrich (Germany). Protein regulation was determined by pixel intensity variance using Carestream Molecular Imaging software (v5.4.2) with Vinculin as an internal standard.

### Reverse transcription and quantitative real-time PCR

Total RNA was extracted from subconfluent monolayers using peqGOLD TriFast (PeqLab, Germany) according to the manufacturer’s instructions. cDNA was transcribed using 2 μg total RNA with the RevertAid First Strand cDNA Synthesis Kit (Fermentas, Germany). cDNA was amplified by RT-PCR using a two-step PCR program of 40 cycles, with denaturation at 95°C for 15 s, annealing and extension at 60°C for 30 s and followed by a melting curve from 50 to 95°C using a Mastercycler ep realplex (Eppendorf, Germany). All primers were synthesized by Sigma-Aldrich (*HPRT*: F:AAGATGGTCAAGGTCGCAAG, R:GTCAAGGGCATATCCTACAACAA; *VEGFA*: F:TACCTCCACCATGCCAAGTG, R:GCTGCGCTGATAGACATCCA, *VEGFA*_*189*_ F:TATAAGTCCTGGAGCGTTCCC, R:CTCGGCTTGTCACATCTGC; *VEGFA*_*165*_: F:AGATAGAGCAAGACAAGAAAATCCC, R:CTCGGCTTGTCACATCTGC, *VEGFA*_*121*_: F:GTGTGTGCCCACTGAGGAG, R:GCCTCGGCTTGTCACATTT, *VEGFR1*: F:CTTCACCTGGACTGACAGCA, R:ACAGCTGGAATGGCAGAAAC; *VEGFR2*: F:ACAACCAGACGGACAGTGGT, R:AGTCAGGCTGGAGAATCTGG; *NRP1*: F:CAAAACCAGCAGACCTGGAT, R:CATTATGCCAACAGGCACAG; *GLUT1*: F:GCTTTGTGGCCTTCTTTGAA, R:CAGAACCAGGAGCACAGTGA). Relative quantification was done using ΔΔCt measurements on SYBR Green based fluorescence readings with HPRT as a housekeeping gene. Measurements were done in triplicate.

### Flow cytometry

Protein expression of receptors on the tumor cell surface was determined by flow cytometry. Cells were harvested using Accutase solution (Sigma-Aldrich, Germany) after 24 hours of normoxia, hypoxia and hypoxia with bevacizumab treatment. Cells were labeled for Neuropilin1 with CD304- and VEGFR2 with CD309-APC conjugated antibodies (Miltenyi Biotec, Germany) and measured by a BD FACS Canto II flow cytometer. HUVEC were used as a control. Analysis was done using FlowJo software (version 8.8.6) to determine the percentages of positive cells. Results represent averaged percentages from two biological repetitions.

Propidium iodide (Sigma-Aldrich, Germany) stained cells were prepared by fixing the cells in 80% ice cold ethanol for up to 48 hours. Cells were then washed with PBS and resuspended and incubated for 30 minutes in 38 mM sodium citrate, 24 μg/ml RNase A and 54 μM propidium iodide prior to FACS measurement.

### Statistical analysis

Unpaired, two-tailed Student’s *t*-test was performed for statistical analysis. A *p*-value of <0.05 was considered to indicate a significant difference.

## Results

### Cell line selection

As VEGFA is thought to work primarily through activation of one of the known VEGF receptors VEGFR1, VEGFR2 and co-receptor Neuropilin1, in general two cell lines per tumor type were selected from the NCI-60 panel of solid tumors (NSCLC: H522, HOP62, CRC: HCT-116, HT-29, KM12, BC: HS-578 T, MDA-MB-231 and one RCC: A498), according to high relative expression levels from publicly available microarray data [26], published data and our own preliminary gene expression data related to angiogenesis pathway genes. These cell lines are also representative of most of the indications where bevacizumab is approved for clinical use and has shown variable efficacy in clinical practice.

### Tumor cell expression of VEGF receptors

**Figure 1:**
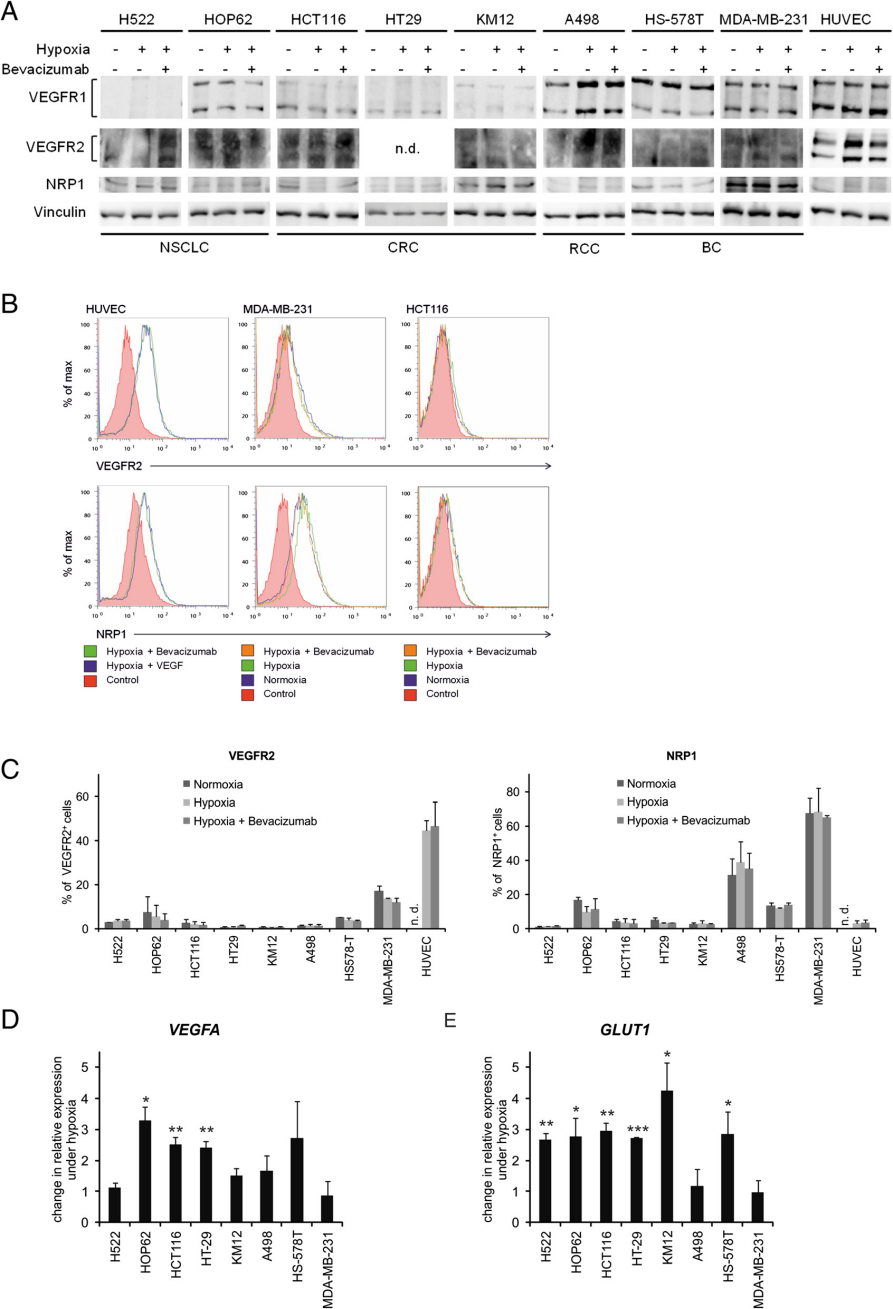
**Expression of VEGF receptors and hypoxic *VEGF *mRNA induction in tumor cells. (A)** Protein expression of VEGFR1, VEGFR2 and NRP1 was determined in tumor cells and HUVECs under normoxia and after 24 hours of hypoxia with or without bevacizumab. Vinculin was used as a loading control. **(B)** Cell surface expression of VEGFR2 and NRP1 as analyzed by flow cytometry. Unstained cells cultured under normoxic conditions were used as a control. **(C)** Quantification of VEGFR2^+^ and NRP1^+^ cell surface expression. **(D)** Relative change of *VEGFA* mRNA expression under hypoxia versus normoxic controls. **(E)** Relative change of *GLUT1* mRNA expression under hypoxia versus normoxic controls. n.d. = not done.

The protein levels of VEGFR1, VEGFR2 and Neuropilin1 expressed by tumor cells were determined by western blot analysis. Total cell lysates from cells treated with or without bevacizumab under hypoxic conditions for 24 hours were examined to determine if there is any regulation of receptor expression compared to normoxic conditions (Figure 1A). The two VEGFR2-specific bands were detected on HUVECs, which was used as a positive control and present in four of the selected tumor cell lines, H522, HOP62 (NSCLC), HCT-116 (CRC) and MDA-MB-231 (BC). Changes in expression of VEGFR2 as result of hypoxia or bevacizumab treatment in tumor cells were difficult to evaluate by western blot, so we therefore assessed transcript changes and localization by flow cytometry. VEGFR1 showed clear expression shown by two bands in all cell lines with the exception of H522 (NSCLC). Whilst hypoxia up regulated expression in A498 (RCC) by 1.8-fold, bevacizumab treatment does not appear to strongly regulate VEGFR1 in the other VEGFR1-expressing cell lines. Interestingly, all cell lines showed expression for Neuropilin1, albeit at varying levels, and protein expression was not significantly altered by either hypoxia or bevacizumab treatment.

### Cell surface expression of VEGF receptors

Although western blot analysis did not show any overall change in expression, to determine if receptor localization was affected by hypoxia or bevacizumab treatment, cell surface localization of VEGFR2 and Neuropilin1 was evaluated by flow cytometry (Figure 1B and 1C and summarized in Table 1). VEGFR1 localization was not analyzed, as no suitable antibody for FACS analysis was available. Although all cell lines showed Neuropilin1 protein expression to varying intensities, this did not necessarily translate to cell surface expression, with no detectable expression on H522, HCT-116, HT-29 or KM12. Neuropilin1 was expressed on the cell surface at a high level in one breast tumor cell line (MDA-MB-231), followed by A498 (RCC). Expression was present to a lesser degree in HOP62 (NSCLC) and HS-578 T (BC) exhibiting approximately 10–15% of cells with receptors at the cell surface.

**Table 1:** Protein and cell surface expression of VEGFR1, VEGFR2 and NRP1

		**NSCLC**		**CRC**			**RCC**	**BC**		
		**H522**	**HOP62**	**HCT-116**	**HT-29**	**KM12**	**A498**	**HS-578 T**	**MDA-MB-231**	**HUVEC**
**Protein expression**	VEGFR1	-	+	-	+	-	+	+	+	+
	VEGFR2	+	-	+	n.d.	-	-	-	+	+
	NRP1	+	+	+	+	+	+	+	+	-
**Cell surface expression**	VEGFR1	n.d.	n.d.	n.d.	n.d.	n.d.	n.d.	n.d.	n.d.	n.d.
	VEGFR2	-	-	-	-	-	-	-	+	+
	NRP1	-	+	-	-	-	+	+	+	-

+ = present in cell line, - = not present in cell line, n.d. = not done.

In contrast to Neuropilin1, VEGFR2 expression was more limited on the surface of tumor cells, in line with western blot analysis. As expected endothelial cells showed expression of VEGFR2 and only one tumor cell line, MDA-MB-231 (BC), with high numbers of VEGFR2 positive cells compared to the other tumor cell lines. The other tumor cell lines that had VEGFR2 protein expression, H522, HOP62 and HCT-116, did not show VEGFR2 on their surface with the percentages of positive cells remaining below 10%. Treatment under hypoxia or with bevacizumab did not influence any obvious change in either Neuropilin1 or VEGFR2 membrane expression.

### Analysis of hypoxic *VEGFA* induction in tumor cells

Activation of HIF-1 under hypoxia should lead to a variety of gene expression changes, including induction of *VEGFA*, which may preferentially trigger specific changes in tumor cells. To this end, cells were incubated under normoxic and hypoxic conditions for 24 hours and total *VEGFA* mRNA levels were measured by quantitative real-time PCR.

Most tumor cells reacted to the hypoxic environment with the induction of either *VEGFA* or *GLUT1* mRNA after 24 hours of hypoxia exposure, however to variable degrees within the different tumor entities (Figure 1D and 1E). Three tumor cell lines had significant induction of *VEGFA,* which did not exactly match the pattern of *GLUT1* mRNA where six cell lines showed significant induction. MDA-MB-231 (BC) and A498 showed no transcriptional regulation of the two classical hypoxia inducible genes whereas KM12 (CRC) and H522 (NSCLC) demonstrated induction of only *GLUT1*. HS-578 T (BC) responded to the hypoxic environment with a 2.7-fold increase of *VEGFA* over the normoxic control and 2.8-fold change for *GLUT1* (p = 0.05). HOP62 (NSCLC) showed the highest induction of *VEGFA* with up to 3-fold (p = 0.012) along all investigated tumor cell lines. For the two colorectal tumor cell lines HCT-116 and HT-29 (CRC) *VEGFA* was upregulated to a similar extent under hypoxic conditions with 2.5-fold (p = 0.008) and 2.4-fold (p = 0.007) (Figure 1D). The change in either *GLUT1* and/or *VEGFA* expression documents the adaptive responsiveness of some tumor cells to the hypoxic environment, giving rise to the possibility of autocrine or paracrine *VEGFA* stimulation in comparison to those cell lines where no induction is evident.

### Gene expression regulation upon bevacizumab treatment

An evaluation of the VEGF signaling molecules was performed to determine if mRNA expression was altered, which may not be apparent by the less sensitive evaluation from protein analysis.

**Figure 2:**
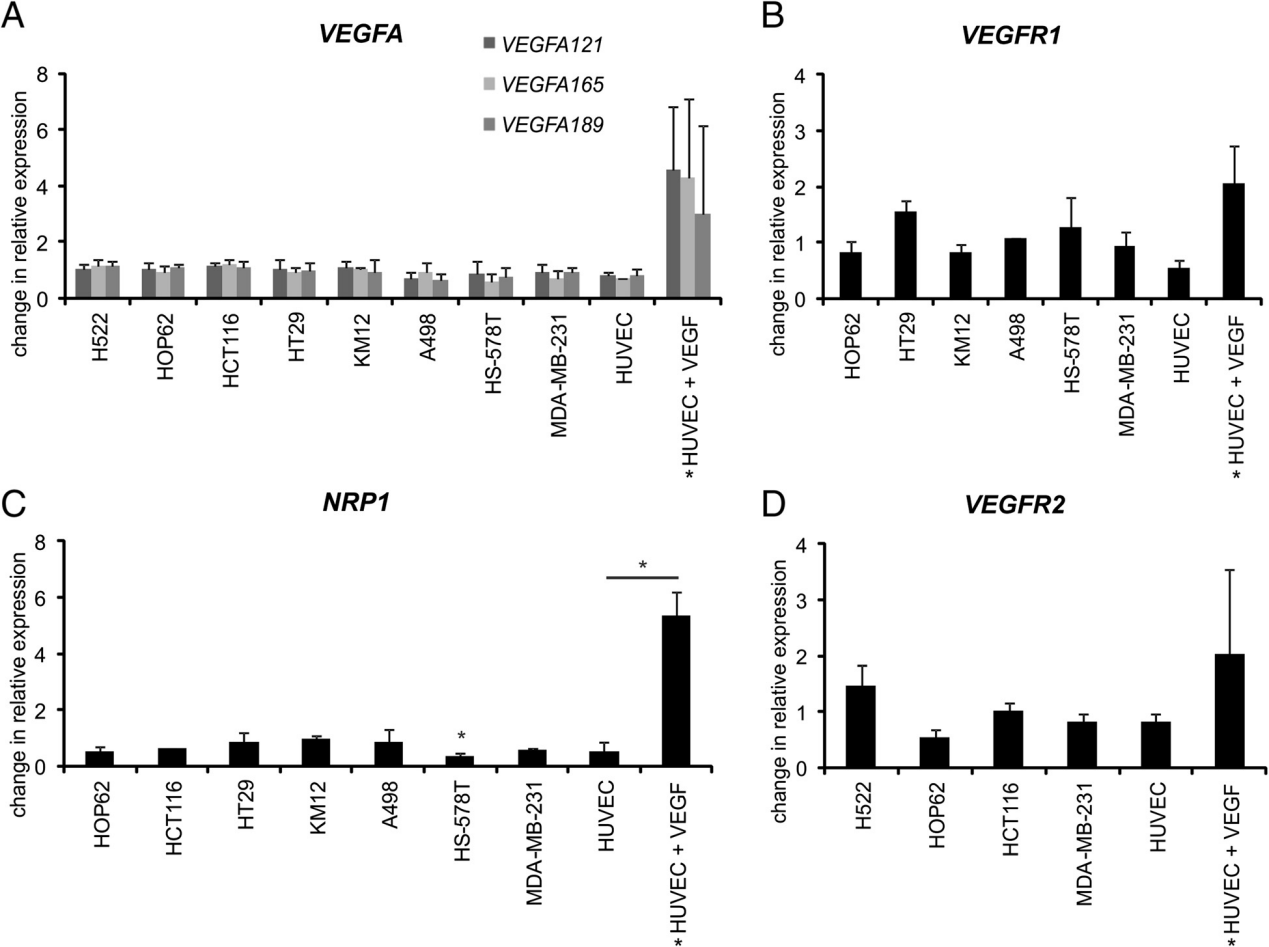
**Gene expression analysis in bevacizumab treated tumor cells.** Change in relative expression of **(A)***VEGFA* isoforms, **(B)***VEGFR1*, **(C)***NRP1* and **(D)***VEGFR2* in bevacizumab treated cells after 24 hours of hypoxia versus untreated hypoxic cells. Only cell lines with detectable expression are included. * indicates HUVECs were in addition stimulated with rhVEGF in the absence of bevacizumab and normalized against untreated controls.

Analysis of the different *VEGFA* isoforms *VEGFA*_121_, -_165_ and -_189_ revealed no evident regulation in all investigated tumor cell lines as well as in HUVECs after bevacizumab treatment in hypoxia for 24 hours. rhVEGF stimulation of HUVECs led to an increase in *VEGFA* isoform expression, however this change was not significant (Figure 2A).

Consistent with the protein analysis, seven cell lines showed *VEGFR1* expression, however there was also no marked change in mRNA levels along with the HUVEC controls (Figure 2 showing only those cell lines with detectable expression). *VEGFR1* was upregulated 2.1-fold in HUVEC when treated with rhVEGF and showed the opposing downregulation of 1.9-fold after rhVEGF and bevacizumab treatment, however downregulation remained below the threshold of significance (Figure 2B). *VEGFR2* was present in four of the cell lines and remained unregulated after 24 hours of bevacizumab treatment in hypoxia in all of the VEGFR2-expressing cell lines (Figure 2D). For HUVECs a 2-fold upregulation of *VEGFR2* was detected after rhVEGF stimulation, but treatment with rhVEGF and bevacizumab only led to a 1.2-fold downregulation, similar to the degree of *VEGFR2* regulation in tumor cells.

The VEGFA co-receptor *Neuropilin1* was significantly decreased in HS-578 T (BC) by a 3-fold down regulation (p = 0.02). The other breast cancer cell line, MDA-MB-231, showed also a downregulation (1.7-fold), however it was below the threshold of significance determined by a 2-fold regulation. HOP62 (NSCLC) and HCT-116 (CRC) demonstrated a downregulation of 1.9- and 1.6-fold after bevacizumab treatment, which also remained below the threshold (Figure 2C). The downregulation was not seen at protein level in either cell line, suggesting perhaps stabilization of proteins or changes in mRNA translation. The remaining cell lines did not exhibit a characteristic pattern of expression or regulation (Figure 2). Interestingly HUVECs, when treated with rhVEGF, showed strong upregulation of *Neuropilin1* and the opposing downregulation when rhVEGF was inhibited by bevacizumab, which is the same pattern of regulation of *NRP1* detected in HS-578 T (BC) (Figure 2C).

In summary, although there is a clear trend towards inhibition of VEGFA induced changes of VEGFA related genes in bevacizumab treated HUVECs, there was no consistent impact on gene expression patterns across the tumor cell lines. Bevacizumab did however significantly alter the *Neuropilin1* expression in HS-578 T along with a clear trend of down regulation in HUVECs and three other cell lines, however not to a significant extent.

### Effects of bevacizumab on tumor cell survival

**Figure 3:**
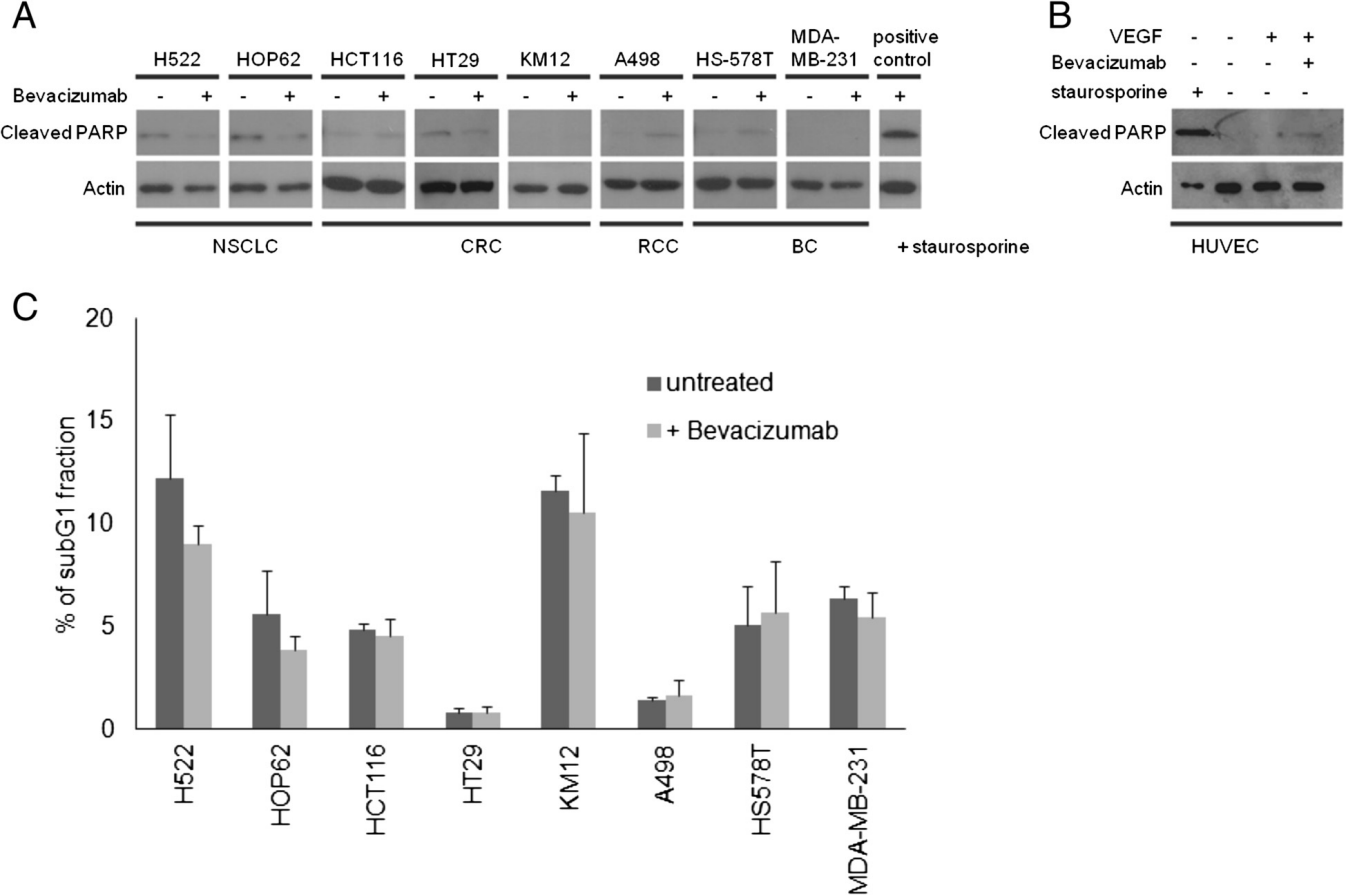
**Tumor cell survival after bevacizumab treatment. (A, B)** Levels of apoptosis were determined in bevacizumab treated cells after 48 hours by western blot analysis using an antibody against cleaved PARP. ß-Actin served as a loading control. As a positive control all cell lines were treated with staurosporine (0.15 μM) for 24 hours to induce apoptosis, an example of KM12 (CRC) and HUVEC is shown. **(C)** Quantification of cellular sub G1 fraction after 48 hours of bevacizumab treatment. Cells were stained with propidium iodide and analyzed by flow cytometry. Averaged data from three experiments are shown.

As VEGFA acts as a survival factor that can rescue endothelial cells from apoptosis, blockade by bevacizumab may have the potential to influence cell survival in tumor cells by the same mechanism. The levels of cleaved PARP (Poly [ADP-Ribose] Polymerase) protein (Figure 3A and 3B) and sub G1 phase propidium iodide conjugated DNA of tumor cell lines (Figure 3C) were taken as indicators of apoptosis. In general, levels of apoptosis were relatively low in all cell lines investigated and they were not further enhanced by bevacizumab treatment under hypoxic conditions with reduced FBS concentrations. Non-small cell lung cancer cells, H522 and HOP62, interestingly showed a decrease in cleaved PARP and sub G1 cells when treated with bevacizumab, however beyond the criteria of significance (p = 0.13 and p = 0.25 respectively). In contrast A498 (RCC) and HS-578 T (BC) exhibited a minor increase in apoptosis according to both cleaved PARP and sub G1 levels. All other cell lines investigated did not show differences after bevacizumab treatment when compared to controls. The magnitude of the effects observed was limited compared to control experiments where each cell line was treated with 150 nM staurosporine for 24 hours as a potent inducer of apoptosis, with a representative example shown for cell line KM12 in Figure 3A.

### Effects of bevacizumab on tumor cell proliferation

**Figure 4:**
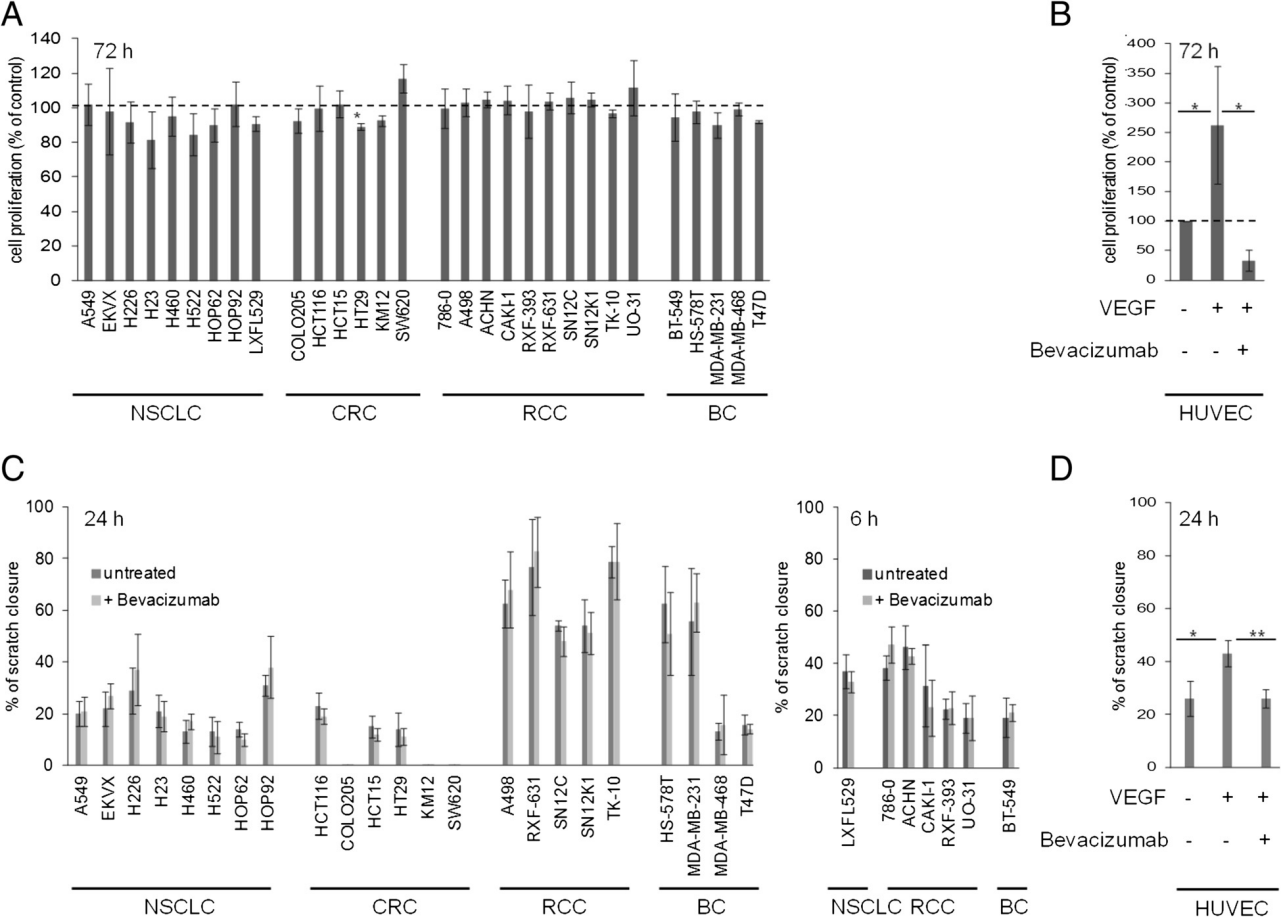
**Tumor cell proliferation and migration analysis after bevacizumab treatment. (A, B)** Proliferation of bevacizumab treated cells as a percentage of control. Cells were cultured under hypoxia and serum starved conditions for 72 hours. For comparison HUVEC were stimulated with rhVEGF and treated with bevacizumab. **(C, D)** Migration of bevacizumab treated versus untreated cells after 24 or 6 hours of hypoxia. HUVEC were again treated with rhVEGF.

With at least one receptor present in the selected cell lines and with the induction of VEGFA under hypoxic conditions, the system was challenged in an effort to reveal an autocrine/paracrine function. Proliferation rates were examined in reduced serum media under hypoxic conditions for up to 96 hours, however overall no obvious change between treated and untreated cells was evident at any of the time points investigated (Figure 4A). Most cell lines did not meet statistical significance according to the student’s 2-tailed t-test, with the exception of HT-29 (CRC).

To determine if an anti-proliferative effect of bevacizumab could be seen in a wider range of cell lines, the analysis was further expanded to include a screen of 30 cell lines from the NCI-60 panel, using the main solid tumor types for which bevacizumab is approved, NSCLC, CRC, RCC and BC. With the exception of HT-29 (CRC) and SW620 (CRC), which showed minor, but opposing changes in proliferation after bevacizumab treatment, an decrease and increase respectively, bevacizumab did not appear to affect tumor cell proliferation. The HUVEC controls did show inhibition of proliferation as expected with bevacizumab (Figure 4B).

In parallel experiments, rhVEGF was added to FBS reduced media in an attempt to stimulate the VEGFA dependent pathways in tumor cells (data not shown). This was however unsuccessful in increasing proliferation rates, including those tumor cells that expressed the major VEGFA signaling receptor VEGFR2 (H522, HOP62, HCT-116, and MDA-MB-231). As a control, HUVECs in contrast did show enhanced VEGF dependent proliferation (Figure 4B).

### Tumor cell migration with bevacizumab treatment

VEGFA has been described as a chemo-attractant and motility factor in endothelial cells, thus blockade of VEGFA by bevacizumab could also influence the migratory potential of tumor cells. Cell migration was assessed by the *in vitro* scratch assay, with experiments performed under hypoxic and serum reduced conditions for all cell lines. Migration was checked after 6 or 24 hours, for cell lines with rapid migration and less motile cell lines respectively (Figure 4C). Migration was highly variable amongst the tumor cell lines, from a complete lack of motility in some colorectal cell lines (COLO205, KM12 and SW620) to complete closure of scratches after 24 hours for five renal cell lines, one lung (LXFL529) and one breast (BT-549) cancer cell line. HUVECs demonstrated a clear dependence on VEGFA for migration with enhanced motility of 1.7-fold, while this effect was reversed by bevacizumab treatment in keeping with previous studies (Figure 4D) [27]. However treatment with bevacizumab was not able to influence the migration of the tumor cells when compared to untreated cells.

## Discussion

VEGFA is a well known and equally well characterized survival factor for endothelial cells [28]. The effect of VEGFA mediated or supported tumor cell proliferation, as a direct effect of the cytokine, is less characterized or established. In line with previous findings, our study demonstrated and confirmed that some tumor cells do harbor VEGF receptors [8,9,13,28,29]. This, coupled with the induction of VEGFA by hypoxia, supports the hypothesis of a possible paracrine or autocrine mechanism that could be disrupted by blocking VEGFA signaling by bevacizumab leading to a direct tumor effect.

It is known that hypoxia is a major regulator of both VEGFA and its receptors [30], however, we found no uniform regulation of receptors or ligands across all cell lines analyzed by either hypoxia or bevacizumab treatment at an mRNA transcript or protein level. Changes detected by mRNA analysis, such as *NRP1* down regulation in HS-578 T (BC), were not translated into protein changes, suggesting alternative regulatory mechanisms, which may be a result of translational variations or post translational modifications along the secretory pathway.

Neuropilin1, which serves as a VEGFA co-receptor, showed some regulation under hypoxic conditions, which is consistent with previous published studies [31]. This effect was however, not uniform across our selected cell lines. Of note, although all cell lines expressed Neuropilin1, cell surface expression of Neuropilin1 appeared to correlate with high co-expression of VEGFR1. Neuropilin1 has been reported to modulate VEGFR1 signaling leading to enhanced migration and survival of VEGFR1 expressing endothelial cells [32]. Three of the four Neuropilin1/high-VEGFR1 expressing cell lines were highly motile, but our migration analysis did not demonstrate any effect of VEGFA depletion via bevacizumab treatment nor in the extended cell line investigation. This may be due to the possibility that migration is controlled through alternative binding partners of VEGFR1, such as VEGF-B or PlGF or apparent after extended bevacizumab exposure for up to 3 month as reported in the study by Fan et al. [33,34]. Migration of tumor cell lines was highly variable and the migration potential was not altered with bevacizumab treatment.

Moreover, tumor cell dependence on VEGFA as a survival factor was explored via the quantification of apoptosis by cleaved PARP and confirmed by FACS analysis, which did not produce evidence that bevacizumab had an effect on cellular survival. It has been shown that depletion of VEGFA or VEGFR1 through knock-down experiments can interfere with the autocrine feedback loop and survival of tumor cells, but only where VEGFR1 is present at nuclear membranes and therefore inaccessible to extracellular ligands or bevacizumab [35]. Our experiments show that the use of a VEGFA targeted antibody is not able to mimic this phenomenon in our cell lines as there is no evidence of a significant increase in apoptotic cells upon single agent treatment.

VEGFA stimulated proliferation induced by hypoxia was not inhibited by bevacizumab treatment and remained more or less unchanged in most tumor cells except HT-29 (CRC). The decrease in proliferation noted in HT-29, could not be attributed to changes in VEGFA related gene or protein regulation and may be related to other downstream components of the HIF response [36]. Small molecule receptor tyrosine kinases targeted to the VEGFA pathway in HT-29 xenografts have shown some tumor cell effects in other studies suggesting this pathway does play a critical role in cell survival, however perhaps only clearly evident when there are multiple receptor targets [37]. The lack of proliferation changes in the other cell lines was consistent at each time point investigated with only minor decreases or increases. In contrast, endothelial cells showed a significant decrease in proliferation rate after bevacizumab treatment. There has been some limited analysis of individual cell lines treated with bevacizumab in the literature, overall concurring with our results of a lack of major effects on proliferation, [38] or even a slight increase in proliferation when treated with bevacizumab alone [39].

The role of VEGFA in generating endothelial cell changes is well established, with the inhibition of VEGFA leading to changes in tumor vasculature [40]. However, patient outcomes using bevacizumab have implied that VEGFA antibodies may also differentially affect the tumor cells or the tumor’s microenvironment. Even with inherent difficulties of *in vitro* studies, our data suggest that tumor cells themselves are not intrinsically affected in an adverse manner by bevacizumab monotherapy based on the selection of assays performed. In addition, the angiogenic potential mediated through the VEGFA pathway was not significantly altered in the tumor cell lines. The effect beyond vasculature permeability, remodeling and pruning of an anti-VEGFA based therapy, is likely to be a complex interaction of tumor vasculature, tumor stroma, immune cells as well as the tumor cells.

## Conclusions

Bevacizumab was the first approved therapeutic agent targeting blood vessels of tumors, which has shown efficacy in suppression of tumor growth and direct evidence of anti-vascular effects in human tumors. The present study showed in a variety of *in vitro* experiments with several tumor cell lines from different tumor origins, that there was a limited measurable effect with bevacizumab monotherapy when evaluating VEGFA induced downstream outputs known from endothelial cells.

## Competing interests

Both authors declare that they have no competing interests.

## Authors’ contributions

Both SG and MH contributed to the study concept, design and data analysis. Both authors have read and approved the final manuscript.
